# Oncometabolite L‐2‐hydroxyglurate directly induces vasculogenic mimicry through PHLDB2 in renal cell carcinoma

**DOI:** 10.1002/ijc.33435

**Published:** 2021-01-15

**Authors:** Huan Wang, Liya Wang, Qiming Zheng, Zeyi Lu, Yuanlei Chen, Danyang Shen, Dingwei Xue, Minxiao Jiang, Lifeng Ding, Jie Zhang, Haiyang Wu, Liqun Xia, Jun Qian, Gonghui Li, Jieyang Lu

**Affiliations:** ^1^ Department of Urology Sir Run Run Shaw Hospital, Zhejiang University School of Medicine Hangzhou China; ^2^ Department of Urology The Affiliated Hangzhou First People's Hospital of Zhejiang University School of Medicine Hangzhou China; ^3^ State Key Laboratory of Modern Optical Instrumentations Centre for Optical and Electromagnetic Research, College of Optical Science and Engineering, Zhejiang University Hangzhou China

**Keywords:** 2‐hydroxyglutarate, epigenetics, oncometabolite, renal cell carcinoma, vasculogenic mimicry

## Abstract

Metabolism reprograming is a hallmark of cancer and plays an important role in tumor progression. The aberrant metabolism in renal cell carcinoma (RCC) leads to accumulation of the oncometabolite l‐2‐hydroxyglurate (L‐2HG). L‐2HG has been reported to inhibit the activity of some α‐ketoglutarate‐dependent dioxygenases such as TET enzymes, which mediate epigenetic alteration, including DNA and histone demethylation. However, the detailed functions of L‐2HG in renal cell carcinoma have not been investigated thoroughly. In our study, we found that L‐2HG was significantly elevated in tumor tissues compared to adjacent tissues. Furthermore, we demonstrated that L‐2HG promoted vasculogenic mimicry (VM) in renal cancer cell lines through reducing the expression of PHLDB2. A mechanism study revealed that activation of the ERK1/2 pathway was involved in L‐2HG‐induced VM formation. In conclusion, these findings highlighted the pathogenic link between L‐2HG and VM and suggested a novel therapeutic target for RCC.

## Abstract 2

**What's new?**

Metabolic reprograming, a hallmark of cancer, influences tumor progression. In the case of renal cell carcinoma (RCC) specifically, progression appears to be facilitated by the oncometabolite L‐2‐hydroxyglurate (L‐2HG), though underlying mechanisms remain enigmatic. Here, the authors investigated the ability of L‐2HG in RCC to promote vasculogenic mimicry (VM), in which aggressive cancer cells form vessel‐like networks that support tumor growth. Analyses of RCC patient tissues revealed elevated L‐2HG levels, wherein tumor cells with greater L‐2HG levels exhibited more VM structures. TCGA data and high‐throughput sequencing analyses further show that L‐2HG contributes to VM formation via reduction of PHLDB2 levels.

**Abbreviations**
ECs: endothelial cells
FPKM: fragments per kilobase of exon per million fragments mapped
GO: Gene Ontology
IHC: immunohistochemistry
KEGG: Kyoto Encyclopedia of Genes and Genomes
L‐2HG: L‐2‐hydroxyglurate
L2HGDH: L‐2HG dehydrogenase
LC‐MS/MS: liquid chromatography‐tandem mass spectrometry
MAPK: mitogen‐activated protein kinase
mRCC: metastatic renal cell carcinoma
OS: overall survival
PAS: Periodic acid‐Schiff
PFS: progression‐free survival
PHLDB2: pleckstrin homology‐like domain family B member 2
RCC: renal cell carcinoma
TCA: trichloroacetic acid
TCGA: the cancer genome atlas
TET: ten eleven translocation
TKI: tyrosine kinase inhibitors
VM: vasculogenic mimicry
α‐KG: alpha‐ketoglutarate

## 1 INTRODUCTION

Renal cell carcinoma (RCC) is responsible for approximately 3.8% of all cancers and 2.5% of all cancer deaths worldwide.^1^, ^2^ Early‐stage RCC is often treated with partial or radical nephrectomy, with a 5‐year survival rate of 92.6%. However, about 25% of RCC patients are diagnosed at the metastatic stage, and a third of the patients who undergo resection of localized tumors will have a recurrence.^3^, ^4^ In recent years, tyrosine kinase inhibitors (TKIs), such as sunitinib and pazopanib, have been administered in clinical treatment, especially in metastatic renal cell carcinoma (mRCC); these drugs significantly prolong the overall survival (OS) rate and progression‐free survival (PFS) time.^5^ However, most patients eventually develop acquired resistance after 6 to 15 months of angiogenesis‐target therapy.^6^, ^7^ Thus, there is an urgent need to investigate the molecular mechanisms of RCC and explore new treatment strategies for it.

RCC is increasingly recognized as a metabolically solid tumor, characterized by dysregulation of cellular energetics and metabolic reprograming.^8^ Previous studies reported that many genes such as *VHL, MET, FH, SDH, FLCN, TSC1* and *TSC2* were involved in pathways responding to metabolic stress.^9^ L‐2‐Hydroxyglurate dehydrogenase (L2HGDH) is an FAD‐dependent enzyme that oxidizes L‐2‐hydroxyglutarate (L‐2HG) to alpha‐ketoglutarate (α‐KG). Interestingly, a recent report provided evidence that reduced mRNA and protein expression of L2HGDH in RCC promoted L‐2HG accumulation and correlated with reduced 5‐hydroxymethylcytosine.^10^ 2HG is a chiral molecule including D‐ and L‐enantiomers. Although IDH1/2 mutants exclusively produce D‐2HG,^11^ L‐2HG is induced by MDH1/MDH2 and LDHA during hypoxia.^12^ L2HGDH and D2HGDH are important enzymes that oxidize 2HG to α‐KG, preventing the accumulation of 2HG.^13^, ^14^ Previous studies demonstrated that both L‐2HG and D‐2HG were elevated in RCC, but L‐2HG was the predominant enantiomer present in RCC, partly due to the low expression of L2HGDH.^10^, ^15^ Due to the structural similarity to α‐KG, 2HG competitively inhibits some α‐KG‐dependent dioxygenases such as ten eleven translocation enzymes (TETs) and the Jumonji family of histone lysine demethylases, which are responsible for DNA and histone hypermethylation.^16^, ^17^ Recently, more evidence showed nonoxygenase enzymes were also implicated, including the DNMT1.^18^ Inhibition of these enzymatic processes played an important role in tumor progression. Furthermore, elevated 2HG levels also resulted in changes to redox metabolism, potentially contributing to an increased risk of cancer.^19^ Previous studies linked the D‐2HG to the leukemia, brain tumors and colorectal cancer progression.^20^, ^21^, ^22^, ^23^ In contrast, Chen et al demonstrated D‐2HG exerted a broad antileukemic activity through FTO/m6A/MYC/CEBPA signaling.^24^ However, more investigations are needed into 2HG‐related targets as well as the effects on tumor progression.

Vasculogenic mimicry (VM) is a new tumor vascular paradigm independent of endothelial cells (ECs), which has emerged as an important vasculogenic mechanism in tumors in addition to classic angiogenesis.^25^ Periodic acid‐Schiff (PAS) staining and CD34 immunohistochemistry (IHC) have been used to evaluate the presence of VM.^26^ VM describes the specific capacity of aggressive cancer cells to form vessel‐like networks, providing adequate nutrition supplements for tumor growth, and it is therefore associated with tumor metastasis and poor prognosis.^27^ VM is identified in various malignant tumors, including breast cancer,^28^ gastric adenocarcinoma,^29^ lung cancer^30^ and prostate cancer.^31^ A systematic review and meta‐analysis of VM, including 3062 patients having 15 types of cancers, showed that VM‐positive patients had a poor 5‐year OS rate compared to VM‐negative patients^32^ and VM drove some tumor cells to distant metastases in human cancers.^33^ In fact, VM was composed of cancer cells, and the mechanism of channel formation was not the same as vessels formed by ECs. In this way, VM was thought to account for resistance to the antiangiogenesis therapy in some tumors.^34^ Therefore, it is of great importance to explore the underlying mechanisms of VM formation so as to deepen the understanding of tumor neovascularization. Key molecular regulators of VM were identified in other cancer types, including MAPK,^35^ vascular endothelial (VE)‐cadherin^36^ and MMPs.^37^ To date, the presence of VM in RCC and its relationship with 2HG have not yet been elucidated.

In our study, we reported a role for L‐2HG in promoting VM formation in RCC through a PHLDB2/MAPK pathway; however, the detailed mechanisms of PHLDB2 associated with RCC need further investigation. Thus, our study adds evidence that the oncometabolite L‐2HG is a potential therapeutic target in RCC.

## 2 MATERIALS AND METHODS

### 2.1 Cell culture and reagents

786‐O (RRID: CVCL_1051), A‐498 (RRID: CVCL_1056), OS‐RC‐2 (RRID: CVCL_1626) and Caki‐1 (RRID: CVCL_0234) cell lines were obtained from the Chinese Academy of Sciences (Shanghai, China). A‐498 and OS‐RC‐2 cell lines authentication was performed using STR profiling within the last 3 years at Shanghai Biowing Applied Biotechnology Co., Ltd. (Shanghai, China). 786‐O and Caki‐1 cell lines authentication was performed using STR profiling within the last 3 years at Zhejiang University Forensic Science Center. All cell lines were routinely tested and shown to be mycoplasma‐free as determined by the polymerase chain reaction (PCR)‐based method. All experiments were performed with mycoplasma‐free cells. A‐498 and OS‐RC‐2 were cultured in Dulbecco's modified Eagle's Med (Invitrogen, Grand Island, NY) supplemented with 10% fetal bovine serum (FBS, Gibco, Australia); 786‐O and Caki‐1 were cultured in Roswell Park Memorial Institute (RPMI) 1640 (Invitrogen, Grand Island, NY) containing 10% FBS. All cells were grown as a monolayer on plastic cell culture dishes at 37°C in a humidified atmosphere containing 5% CO_2_.

L‐2HG octyl esters (HY‐103641A), decitabine (HY‐A0004) and U0126 (HY‐12031) were purchased from the MedChemExpress (MCE, China). Diacetyl‐L‐tartaric anhydride (DATAN, 358924), L‐2HG (90790) and R‐2HG (H8378) were purchased from Sigma‐Aldrich (St. Louis).

### 2.2 siRNA and plasmid construction

PHLDB2 siRNAs and a negative control were purchased from RiboBio (Guangzhou, China) and the target sequences were (1#) CACCAGGAATGATGAACTA and (2#) CAGCGAGTCCTCTTATCTA. Lipofectamine RNAiMAX (Invitrogen, America) was used for siRNA transfection. GV141‐L2HGDH and GV657‐PHLDB2 plasmid were designed and synthesized by GeneChem (Shanghai, China). Transfection was performed using Lipofectamine 3000 (Invitrogen, Carlsbad, CA) when cells were at 50% confluence according to the manufacturer's instructions.

### 2.3 L‐2HG treatment and measurement

L‐2HG octyl esters are commonly used in place of L‐2HG because they can permeate across the cell membrane more easily and can be converted by intracellular esterase to 2HG.^10^ For effects on phenotypes in RCC cell lines, cells were continuously cultured in the indicated concentration of L‐2HG for 2 days.

L‐2HG analysis of samples was performed in Medsyin Biotec Co, Ltd (Shanghai, China). Briefly, for measurement of L‐2HG, tissues or cells were washed in cold phosphate‐buffered saline (PBS) three times. Then, total metabolite was extracted with 10% trichloroacetic acid (TCA) and the precipitate was removed by centrifugation. TCA in the supernatant was removed by vortexing with four volumes of 1,1,2‐trichlorotrifluoroethane‐trioctylamine mixture and the upper aqueous layer was collected after centrifugation.

L‐2HG and R‐2HG derivatization was consistent with previous studies.^38^, ^39^ Briefly, extracts were derivatized with DATAN, enabling the separation of the enantiomers (Figure S1A,S1B). The extracts were dried under N_2_ at 35°C, after which 100 μL of 50 g/L of DATAN was added (dissolved in dichloromethane: acetic acid, 4:1, v/v). The reaction proceeded for 30 minutes at 75°C, and then the mixture was dried under N_2_ at room temperature. When completely dry, the mixture was redissolved in 200 μL of water and subjected to the liquid chromatography‐tandem mass spectrometry (LC‐MS/MS, Agilent 6460). The amount of L‐2HG and R‐2HG in extracts was quantified by using a calibration curve and normalized to protein content.

### 2.4 Patients and tissue specimens

We used 27 pairs of tumor and adjacent tissue samples pathologically diagnosed with RCC from Sir Run Run Shaw Hospital who were in our study. All patients' clinical and follow‐up information was complete and available between 2016 and 2019, and all samples were collected with informed consent according to the Internal Review and the Ethics Board of Sir Run Run Shaw Hospital (IRB number 20190211‐67). The patients' characteristics are presented in Table 1.

**TABLE 1:** Patient characteristics

Variables	Case number (N%) or mean(range)
Gender	
Male	21 (77.78%)
Female	6 (22.22%)
Age (years)	60.18 (37‐84)
Tumor stage	
I	21 (77.78%)
II	5 (18.52%)
III or IV	1 (3.7%)
Fuhrman grade	
1–2	14 (51.85%)
3‐4	13 (48.15%)

### 2.5 Tube formation assay and quantification

786‐O, A‐498 and OS‐RC‐2 cells were harvested then resuspended in serum‐free DMEM at 3 × 10^5^/mL. Growth factor‐reduced Matrigel (BD Bioscience) at 40 μL was plated to 96‐well plates at a horizontal level that allowed the Matrigel to distribute evenly and incubated for 0.5 hour at 37°C. Then 100 μL of resuspended renal cancer cells was added on top of the Matrigel. Each condition had at least three replicates. After incubation at 37°C for 24 hours, each well was analyzed under a microscope. The structures with at least half tubular connection were counted as being positive. Tubule numbers in each field were imaged and the average of tubules counted from three random fields in each well was calculated.

### 2.6 Quantitative PCR

Total RNA was extracted from RCC patient samples and RCC cell lines using the RNA‐Quick purification kit (ES‐RN001, Shanghai Yishan Biotechnology, China) according to manufacturer's recommended protocol. Then 500 ng of total RNA was subjected to reverse transcription using the Easy Quick RT Master Mix kit (CW2019, CWBIO, China). The qRT‐PCR was conducted using the Light Cycler 480 instrument (Roche Diagnostics) with Ultra SYBR Mixture (CW0957, CWBIO, China) to determine the expression levels of mRNAs. Expression levels were normalized to the expression of β‐actin. The relative fold change was calculated by the 2‐ΔΔCt method. Primer sequences were listed as follows:

PHLDB2 (F): CCAGGGAACGGGAAATGGAA

PHLDB2 (R): GGTAGCGTGTCAAAGGACGA

ZBTB38 (F): TGTCTTGAAGTGAGGCTCTGCTG

ZBTB38 (R): AGCAAGCCTTGTGGACCAAAC

SIK1(F): AGCTGCTGTTCTGTAGAGAC

SIK1(R): ACGGGCACTTGAGTGAAAAC

FAM3C (F): GGAACATACGATGATGGAGCAAC.

FAM3C (R): GGAACATACGATGATGGAGCAAC

AHR (F): GTCGTCTAAGGTGTCTGCTGGA

AHR (R): CGCAAACAAAGCCAACTGAGGTG

SHIS5A (F): GAAAGGTGTGCTGTGCCTGA

SHIS5A (R): TGACATGGGGTCGTTGTAGC

IGFBP3 (F): GGTCCCTGCCGTAGAGAAAT

IGFBP3 (R): GGCTGCCCATACTTATCCAC

ITPR1 (F): GTGACAGGAAACATGCAGACTCG

ITPR1 (R): CAGCAGTTGCACAAAGACAGGC

GORASP1 (F): GAGGTGGAGAACTCGGTATTGC

GORASP1 (R): AGGTCCATCGTGGCTCCTTTAG

### 2.7 IHC and PAS staining

The tumor tissues were fixed in 4% neutral‐buffered paraformaldehyde, embedded in paraffin, cut into 5‐μm sections and used for IHC. In brief, the tissues were deparaffinized and rehydrated, and the samples were subjected to citrate‐mediated high‐temperature antigen retrieval, then incubated overnight with the primary antibodies CD34 (bs‐0646R, Bioss, China) at 4°C. The sections were rewarmed, washed with PBS and then incubated with goat anti‐rabbit secondary antibody for 30 minutes at room temperature. The sections were then slowly washed with PBS, and peroxidase activity was visualized with diaminobenzidine using the protocol supplied by the manufacturer. To detect VM structures, a PAS staining kit (BASO BA‐4080A) was applied before hematoxylin counterstaining. VM numbers were counted from three randomly chosen fields.

### 2.8 Western blot

Cells were washed with cold PBS twice and were lysed in Cell Lysis Buffer for Western (P0013, Beyotime, China) for 30 minutes on ice. Homogenized samples were then centrifuged for 15 minutes at 13000*g* at 4°C and the supernatant was collected for Western blotting. After that, the proteins (20 μg) were separated on 8% to 12% SDS/PAGE gel and then transferred onto PVDF membranes (Millipore, Billerica, MA). After blocking with 3% bovine serum albumin (BSA) for 1 hour at room temperature, they were incubated with primary antibodies overnight at 4°C. On the next day, anti‐mouse or anti‐rabbit IgG secondary antibodies (Cell Signaling Technology) were used for 1 hour at the concentration of 1:5000 at room temperature and the bands were visualized using the ECL chemiluminescent detection system (Thermo Fisher Scientific). The following antibodies were used: anti‐GAPDH (ab181602, abcam, 1:5000), anti‐MMP9 (10375‐2‐AP, Proteintech, 1:500), anti‐ERK1/2 (ab17942, abcam, 1:1000), anti‐phosphor‐ERK1/2 (ab151279, abcam, 1;1000), anti‐vimentin (5741, CST, 1:1000), anti‐AKT (4691S, CST, 1:1000) and anti‐phospho‐AKT (4058S, CST, 1:1000).

To detect another protein in the same membrane, Stripping Buffer (FD0050, Hangzhou FUDU Biological Technology Co. Ltd, China) was used because it could remove the primary and secondary antibodies from the membranes without affecting the bound antigen. In brief, membranes were fully immersed in a suitable volume of Stripping Buffer at room temperature for 30 to 60 minutes. Then membranes were washed with tris‐buffered saline with Tween‐20 (TBST) three times, 5 minutes each. After blocking the membranes with 3% BSA for 1 hour at room temperature, another antibody was incubated and detected.

### 2.9 mRNA high‐throughput sequencing analysis

Total RNA from the cells was isolated with TRIzol, and mRNAs were purified using oligo‐dT magnetic beads according to the manufacturer's protocol. Subsequently, the enriched mRNAs were transcribed into cDNA and amplified using a random priming method. Then, the RNA libraries were sequenced using the Illumina HiSeq4000 at Shanghai Genesky Biotechnologies Inc, China. The RNA‐seq sequence reads were aligned to the reference genome using GRCh37 (hg19). Quantitative expression of genes and transcripts as fragments per kilobase of exon per million fragments were mapped (FPKM) by the Stringtie analysis process. Differential expression analysis was performed using the DESeq2 R package.^40^ Genes with *P* value less than .05 and |log2 (fold change) | greater than 1 were set as the thresholds for significantly differential expression. Hierarchical clustering was performed to show the distinguishable gene expression pattern among samples.

### 2.10 Survival and methylation analyses with TCGA data

To predict and clarify the prognostic functions of PHLDB2 in RCC, we collected survival data from The Cancer Genome Atlas (TCGA). A total of 517 RCC patients in the TCGA database were enrolled in our study and their information is provided in Supplementary Table 1. Kaplan‐Meier analysis was performed on the UCSC Xena platform.^41^ We also explored the DNA methylation and gene expression in the Wanderer by the TCGA methylation arrays (450K Infinium chip).

### 2.11 Statistical analysis

Data were expressed as the means ± SDs. All statistical analyses were performed using the statistical software (SPSS 24.0, SPSS, Inc.) or GraphPad Prism 7 (GraphPad Software, Inc., CA). Statistical analyses were performed with the Student's *t* test and one‐way analysis of variance (ANOVA) test; *P* values less than .05 were considered significant.

## 3 RESULTS

### 3.1 L‐2HG was elevated in RCC and promoted VM in RCC

**FIGURE 1:**
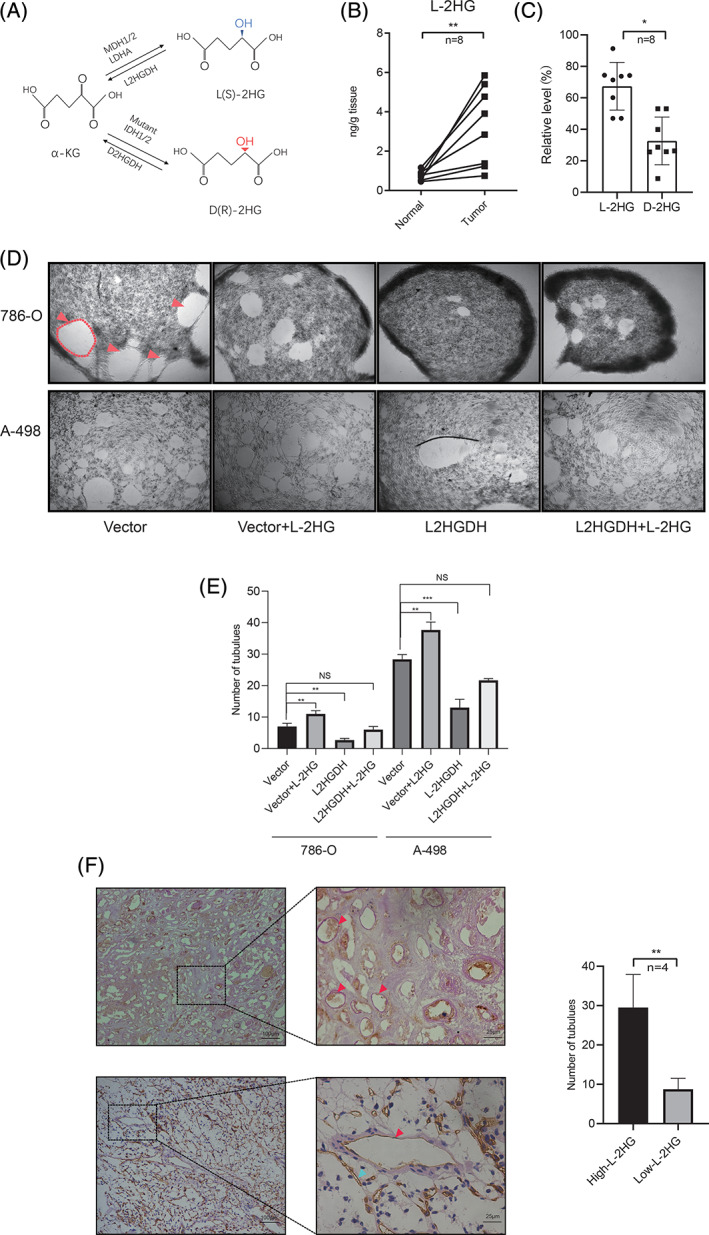
L‐2HG was elevated and promoted VM in RCC. A, The metabolic pathway of the production of D‐ and L‐2HG. α‐KG was metabolized to D‐2HG by mutant IDH1 or IDH2, or to L‐2HG by LDHA or MDH. Furthermore, D‐2HG was converted physiologically to α‐KG by D2HGDH, and L‐2HG to α ‐KG by L2HGDH. B, L‐2HG level was measured by LC‐MS/MS in the RCC tissues and adjacent normal tissues, n = 8, ** *P* < .01. C, Relative ratio of L‐2HG and R‐2HG against total 2HG in tumor tissues was measured by LC‐MS/MS. n = 8, *P < .05. D, Images of VM in 786‐O and A‐498 cells after L‐2HG treatment or L2HGDH plasmid transfection. (Top) Images were taken (×40 magnification) 24 hours after seeding on the Matrigel surface. The representative VM is outlined with red lines. The red arrows indicate VM channels. (Bottom) VM was quantified from three repeat experiments. ** *P* < .01, ***P < .001, NS = no significance. E, The presence of VM in tumor tissues (high L‐2HG vs low L‐2HG). (Left) Red arrows indicate VM channels (PAS positive and CD34 negative), which were stained pink. Scale bars = 100 μm. (Right) Quantification of tubes was determined by microscopy with ×40 magnification in randomly chosen fields. Data are presented as means± SD. n = 4, **P < .01 [Color figure can be viewed at wileyonlinelibrary.com]

D‐2HG and L‐2HG have different metabolic pathways (Figure 1A). To investigate the role of the oncometabolite L‐2HG in RCC, we first demonstrated the L‐2HG level as previously described.^10^ After derivatization by DATAN, L‐2HG and R‐2HG levels could be determined by LC‐MS/MS. (Figure S1A,S1B). Consistent with the previous study,^10^ we also observed high levels of L‐2HG in tumor tissues compared to adjacent tissues (*P* < .01, Figure 1B), while R‐2HG did not increase significantly (*P* > .05, Figure S1C). In addition, L‐2HG was the dominant enantiomer in tumor tissues and at a level about 3‐fold more than R‐2HG (*P* < .05 Figure 1C). When seeded on the Matrigel surface, RCC cells tended to form loops and networks as shown by the red arrows in Figure 1D. The vessel‐like structures formed by the tumor cells are called VM; the process is independent of angiogenesis and is associated with tumor progression.^42^ Thereafter, we examined the effect of L‐2HG on the VM in three typical RCC cell lines (786‐O, A‐498 and OS‐RC‐2), by altering the L‐2HG level. As shown in Figure 1D and Figure S1E, L‐2HG octyl ester treatment significantly promoted VM formation in 786‐O, A‐498 and OS‐RC‐2 cell lines (Vector vs Vector+L‐2HG, **P < .01). On the other hand, reducing the L‐2HG cellular level via overexpressing L2HGDH suppressed VM formation (Vector vs L2HGDH, **P < .01, ***P < .001). More importantly, L‐2HG exposure did not further promote VM after overexpressing the L2HGDH (Vector vs L2HGDH + L‐2HG, NS = no significance). In contrast, R‐2HG did not show the promotion of VM in 786‐O and A‐498 cells (Figure S1D). Furthermore, VM structures were formed by tumor cells, so VM could be examined by double staining of the endothelial cell marker CD34 and PAS. The VM structure criteria were (a) vascular‐like channels lined with tumor cells and (b) positive for PAS but negative for CD34 (PAS + /CD34 −, red arrows in Figure 1E). Interestingly, both angiogenesis and VM presented commonly in RCC. However, the tumors with high L‐2HG levels exhibited more VM structures than those with low L‐2HG levels (*P* < .05, Figure 1E and Figure S1F). Collectively, both in vitro and in vivo data suggested that L‐2HG promoted VM in RCC.

### 3.2 Transcriptome analysis revealed the expression profile of RCC cells after L‐2HG treatment

**FIGURE 2:**
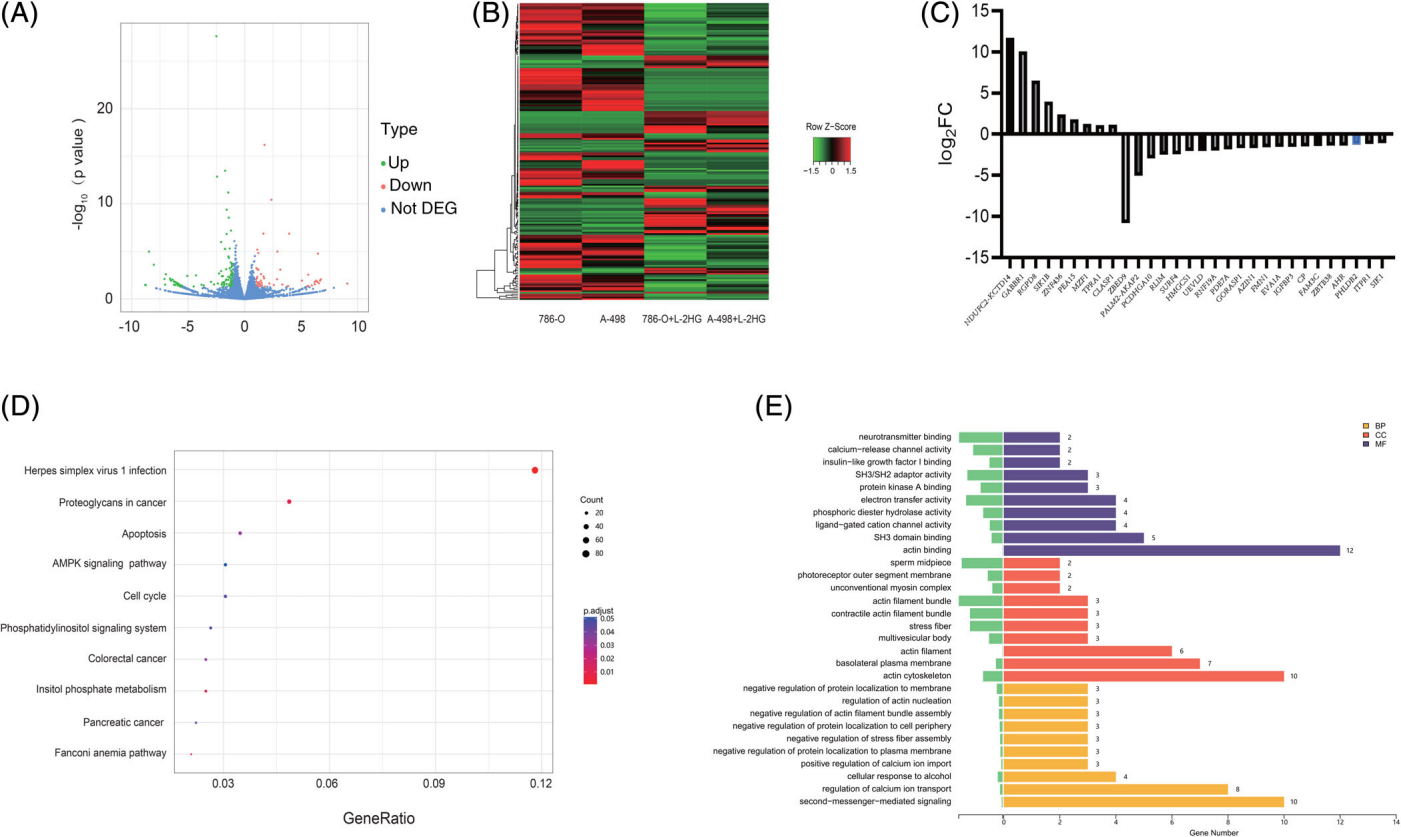
Enrichment of VM‐related genes after L‐2HG treatment. A Volcano plot shows the different expressed genes (DEGs) after L‐2HG treatment in both 786‐O and A‐498 cell lines with the criteria *P* < .05 and |fold change| ≥ 2. Red: upregulated genes, green: downregulated genes. B, Heat‐map overview of the DEGs. C, Average relative expression of the top 20 DEGs compared to the control group in the 786‐O and A‐498 cell lines. D, Dot plot ranking of the *P* values for the significant enrichment pathways according to KEGG analysis of DEGs between the control and L‐2HG treatment groups, *P* < .05. E, Bar plot ranking the gene number according to the GO analysis of DEGs between the control and L‐2HG treatment groups, *P* < .05 [Color figure can be viewed at wileyonlinelibrary.com]

**FIGURE 3:**
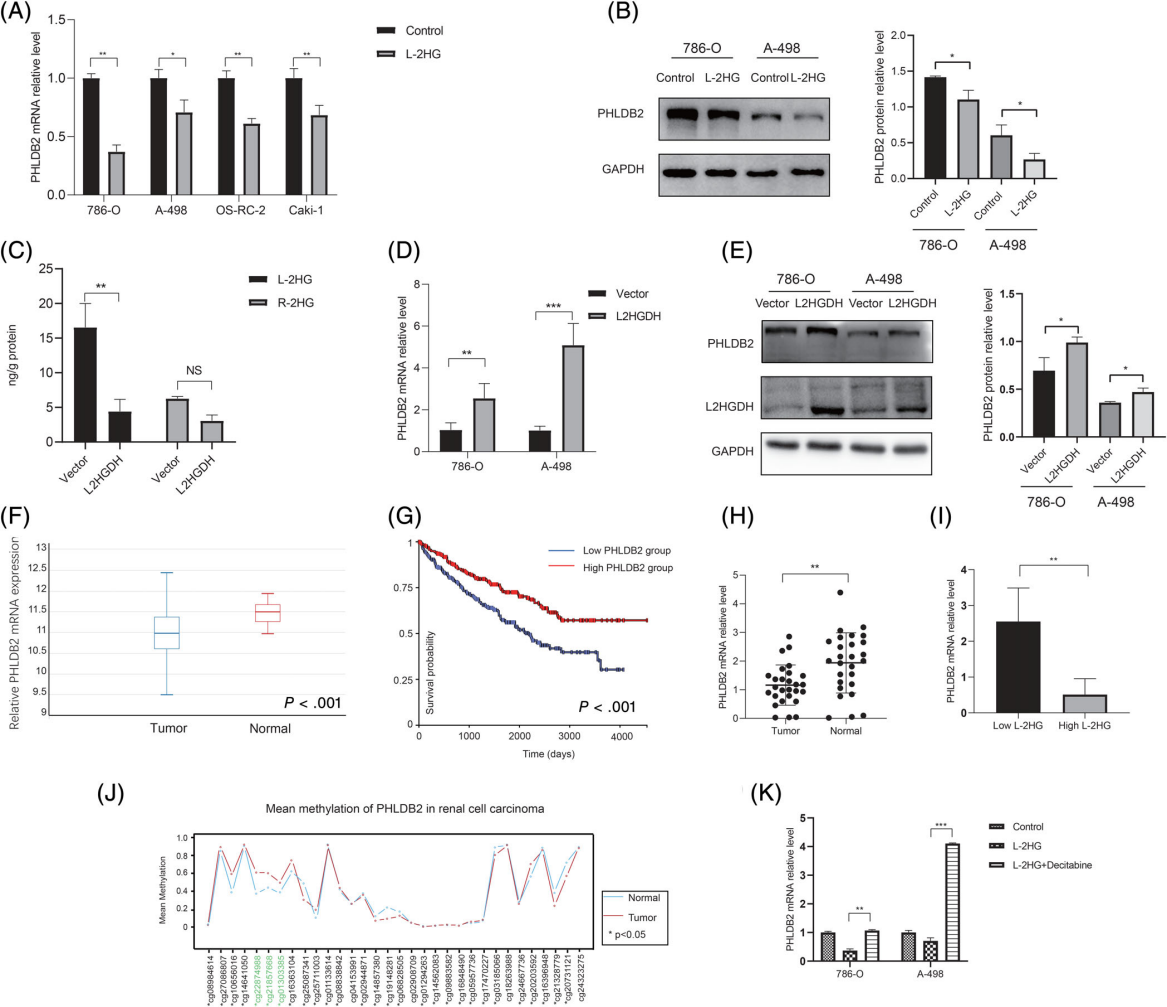
PHLDB2 was downregulated by L‐2HG and associated with progression in RCC. A, Relative PHLDB2 mRNA expression was measured via qPCR after L‐2HG treatment in renal cancer cell lines, including 786‐O, A‐498, OS‐RC‐2 and Caki‐1. GAPDH was used as the internal control. *P < .05, **P < .01. B, Relative PHLDB2 protein expression was measured via Western blot after L‐2HG treatment in 786‐O and A‐498. GAPDH was used as the internal control, one corresponding loading control has been shown. Quantification data based on the bands' density from three independent experiments are on the right panel. C, LC‐MS/MS analysis of L‐2HG and R‐2HG levels in control and L2HGDH expressing 786‐O cells. D, Relative PHLDB2 mRNA expression level was measured via qPCR after vector and L2HGDH plasmid transfection. **P < .01, ***P < .001. E, Relative PHLDB2 protein expression was measured via Western blot after vector and L2HGDH plasmid transfection in 786‐O and A‐498, one corresponding loading control has been shown. Quantification data based on the bands' density from three independent experiments are on the right panel. F, The expression of PHLDB2 in tumor and normal tissues from the TCGA database is presented as means ± SD, n = 517. *P* < .001. G, Based on the expression of PHLDB2, all RCC patients from the TCGA database were divided into two groups (high PHLDB2 group and low PHLDB2 group). Overall survival of RCC patients was associated with the PHLDB2 expression. n = 517, *P* < .001. H, The expression of PHLDB2 in tumor and adjacent tissues from the Sir Run Run Shaw Hospital were measured via qPCR. n = 27, **P < .01. I, According to L‐2HG level, patients were divided into two groups (high L‐2HG group and low L‐2HG group). PHLDB2 expression in the two groups is shown. **P < .01. J, Methylation levels of *PHLDB2* in 30 pairs of RCC tissues and normal tissues were determined by 450K methylation array based on the TCGA data. The three methylation positions labeled with green were associated with the expression of PHLDB2. *P < .05. K, PHLDB2 mRNA expression in 786‐O and A‐498 cells after decitabine (0.5 μM) treatment or L‐2HG treatment. **P < .01, ***P < .001 [Color figure can be viewed at wileyonlinelibrary.com]

To gain mechanistic insights into VM formation by L‐2HG, we compared global gene expression profiles of RCC cell lines with and without L‐2HG treatment cells (786‐O + L‐2HG vs 786‐O and A‐498 + L‐2HG vs A‐498) using RNA‐seq analysis(Supplementary Table 2). In total, 187 differentially expressed genes (DEGs) including 62 upregulated genes and 125 downregulated genes (|fold change| ≥ 2, *P* < .05) were found (Figure 2A). A heat map of the DEGs is presented in Figure 2B, and the average fold change of top 20 DEGs in 786‐O and A‐498 cell lines is shown in Figure 2C. Gene Ontology (GO) and Kyoto Encyclopedia of Genes and Genomes (KEGG) were used to investigate the biological functions of these DEGs. Functional profiling of the 187 DEGs suggested that the affected genes were enriched in biological processes including proteoglycans in cancer, apoptosis, cell cycle and AMPK signaling pathways (Figure 2D). Importantly, GO function classification includes processes such as actin binding, actin cytoskeleton and second messenger‐mediated signaling (Figure 2E). We focused on the top 20 DEGs that were upregulated or downregulated in the two groups (Figure 2C). These genes were further demonstrated by quantitative PCR (qPCR) in the 786‐O group (786‐O + L‐2HG vs 786‐O). We obtained three candidate genes (PHLDB2, ZBTB38 and SIK1), consistent with the RNA‐seq results that had changed most obviously (Figure S2A). In addition, reducing the L‐2HG level by overexpressing L2HGDH reversed these genes' expression (Figure S2B). These results suggested that PHLDB2, ZBTB38 and SIK1 were downregulated by L‐2HG. Analysis of these genes in the RCC using the TCGA data implied PHLDB2 expression was associated with the survival of RCC patients. Therefore, PHLDB2 was the focus of our study, while ZBTB38 and SIK1 functions will need further investigations in the future. More importantly, PHLDB2 changed significantly upon L‐2HG treatment in the other three RCC cell lines (A‐498, OS‐RC‐2, Caki‐1, Figure 3A, *P < .05, **P < .01). In addition, Western blot analysis showed that L‐2HG treatment induced a significant decrease of PHLDB2 level in cells (Figure 3B, *P < .05), and overexpressing L2HGDH significantly reduced the L‐2HG level without affecting the R‐2HG level in 786‐O cells (Figure 3C, **P < .01, NS = no significance). Furthermore, PHLDB2 mRNA and protein levels were both reversed by L2HGDH overexpression (Figure 3D,E). In summary, these results indicated that PHLDB2 was downregulated by L‐2HG.

### 3.3 PHLDB2 was downregulated by L‐2HG and associated with progression in RCC

To investigate the regulatory mode of the VM formation by PHLDB2, we analyzed the relationship between PHLDB2 expression level and the OS rate on the UCSC Xena platform, including 517 RCC patients from the TCGA database. The results indicated that PHLDB2 was downregulated in tumor tissues compared to adjacent tissues and the expression level of PHLDB2 was negatively associated with the OS rate (*P* < .001, Figure 3F,G). In our own tissue samples from Sir Run Run Shaw Hospital, we also observed downregulation of PHLDB2 in tumor tissues compared to normal tissues (**P < .01, Figure 3H). Furthermore, the tumors with high L‐2HG levels were correlated with low expression of PHLDB2 (**P < .01, Figure 3I).

Previous studies indicated that L‐2HG induced the DNA methylation by inhibiting the TETs' function.^10^, ^43^ Therefore, we analyzed whether low expression of PHLDB2 was caused by the DNA methylation. In analysis of the TCGA data in the Wanderer,^44^ the methylation levels of *PHLDB2* promoter regions were significantly different between tumor and normal tissues (Figure 3J). More, cg01303385, cg21857668 and cg22874988 were associated with the downregulation of PHLDB2 (Table 2 and Figure S3A, S3B and S3C). In addition, high cg21857668 levels correlated with poor OS in RCC patients based on the TCGA data while cg01303385 and cg22874988 showed no significance (Figure S3D, S3E and S3F). Furthermore, we treated the RCC cell lines with decitabine (0.5 μM), a DNA methyltransferase inhibitor, and confirmed methylation changes affected the PHLDB2 expression (Figure 3K). Overall, these results suggested that PHLDB2 played an important role in RCC progression and was regulated by L‐2HG.

**TABLE 2:** Methylation of PHLDB2 in RCC (n = 324, 450K methylation array)

Probe ID	Correlation with PHLDB2 mRNA (Pearson)
cg08984614	−0.077
cg27096807	0.038
cg10656016	−0.214
cg14641050	−0.104
**cg2287498**	**−0.421** *
**cg21857668**	**−0.444** *
**cg01303385**	**−0.447** *
cg16363104	−0.125
cg25087341	−0.307
cg2571003	−0.196
cg01133614	−0.182
cg08838842	−0.335
cg02944871	−0.222
cg14857380	−0.003
cg19148281	−0.048
cg06828505	−0.093
cg02908709	0.135
cg1294263	−0.028
cg14562083	0.026
cg09883582	−0.243
cg16848490	0.007
cg05957736	−0.13
cg17470227	0.109
cg03185066	−0.153
cg18263988	−0.087
cg24667736	−0.358
cg20203592	−0.116

^*^ *P* < .05.

### 3.4 PHLDB2 was associated with VM formation through MAPK pathway

**FIGURE 4:**
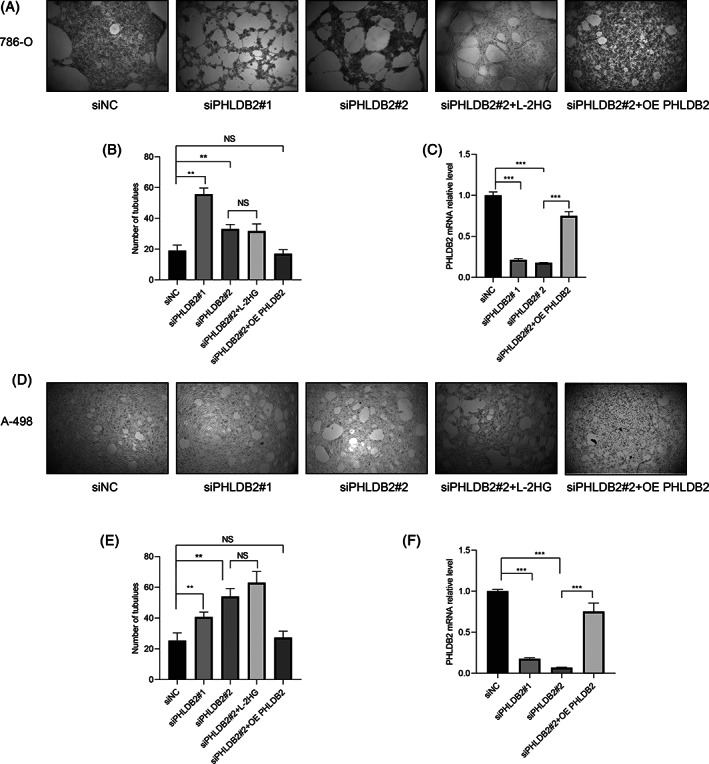
PHLDB2 was associated with VM. A,D, VM formation assays were performed in 786‐O and A‐498 cells with siNC (negative control), PHLDB2 siRNA#1, PHLDB2 siRNA#2, PHLDB2 siRNA#2 + L‐2HG and PHLDB2 siRNA#2 + OE (overexpression) PHLDB2. B,E, Quantification of tubes was determined by microscopy with ×40 magnification in three randomly chosen fields. Data are presented as means ± SD. **P < .01, NS = no significance. C,F, PHLDB2 mRNA relative level was determined after siRNA or PHLDB2 plasmid transfection for 48 hours.***P < .001. A‐C were 786‐O cells. D‐F were A‐498 cells

**FIGURE 5:**
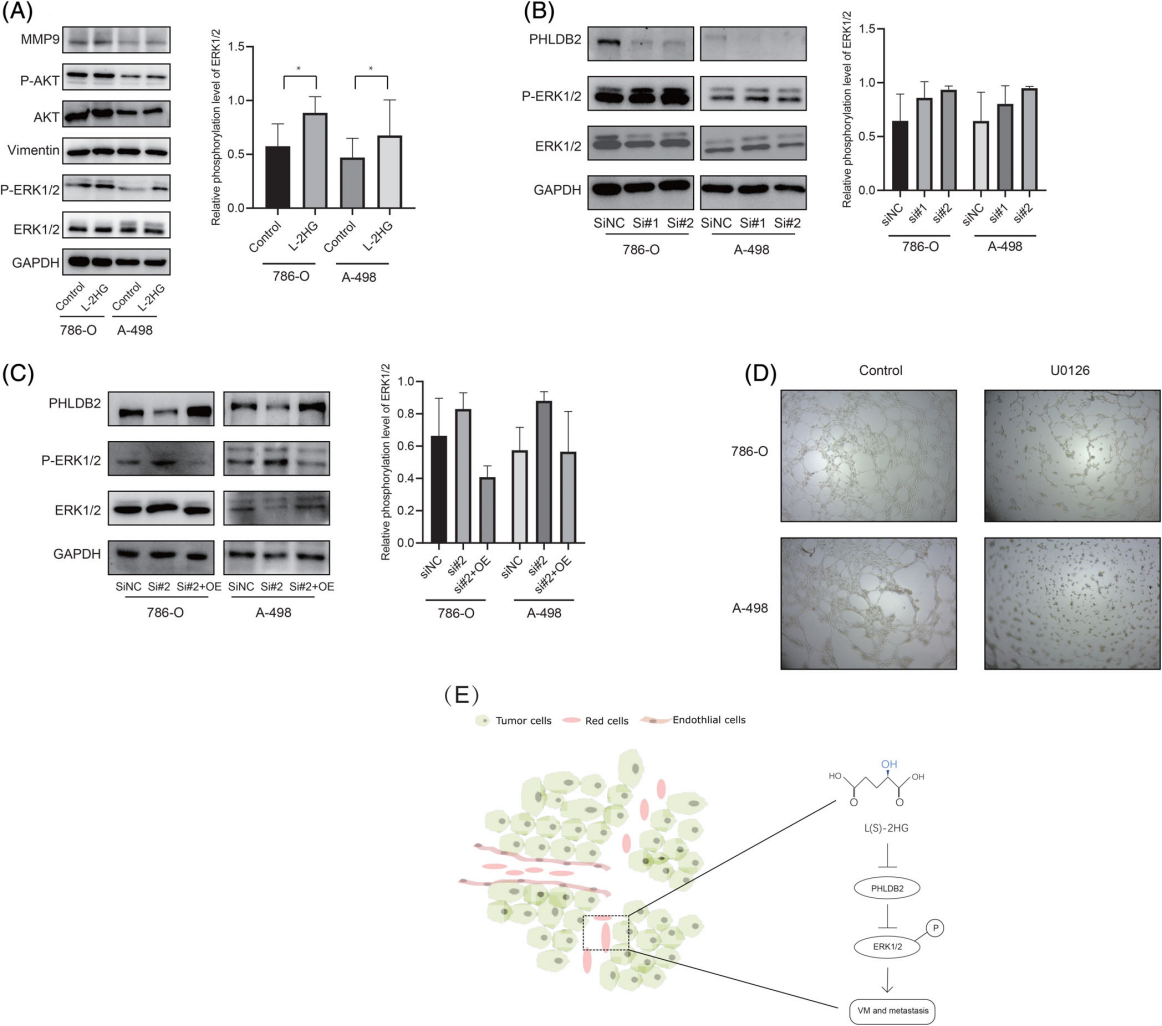
L‐2HG induced VM formation via PHLDB2/ERK pathway. A, Western blot assay for the VM‐related genes expression after L‐2HG treatment in 786‐O and A‐498 cells. One corresponding loading control has been shown. Semiquantification of P‐ERK1/2 based on band density from three independent experiments was shown in the right panel. * *P* < .05. B, Western blot assay for comparing ERK1/2 phosphorylation in 786‐O and A‐498 cell lines after transfection siPHLDB2#1 or #2. One corresponding loading control has been shown. Semiquantification of P‐ERK1/2 based on band density from three independent experiments was shown in the right panel. C, Western blot assay for comparing siNC, siRNA#2 and siRNA#2 + OE PHLDB2. One corresponding loading control has been shown. Semiquantification of P‐ERK1/2 based on band density from three independent experiments was shown in the right panel. D, Images of VM formation after U0126 (1 μM) treatment for 24 hours in 786‐O and A‐498 cell lines. E, Schematic model illustrating the mechanism by which L‐2HG promotes VM and the aggressiveness of RCC [Color figure can be viewed at wileyonlinelibrary.com]

It was reported that PHLDB2 (also known as LL5β), a PH domain‐containing protein, played an important role in mediating cell migration by forming a complex with its partners, such as CLASPS and Prickle 1.^45^, ^46^ However, whether and how PHLDB2 was implicated in RCC progression remained largely unknown. In our study, we found that PHLDB2 at both mRNA and protein levels was downregulated by L‐2HG (Figure 3A,B). Next, we investigated the role of PHLDB2 in L‐2HG‐induced VM formation. After reducing PHLDB2 using two different siRNAs (siPHLDB2#1 and siPHLDB2#2), VM formation increased significantly in 786‐O cells (**P < .01; Figure 4A‐C). In PHLDB2 silenced cells, L‐2HG exposure did not further promote VM formation (siPHLDB2#2 vs siPHLDB2#2 + L‐2HG, NS = no significance). More importantly, VM formation was reversed after restoring the PHLDB2 expression (siPHLDB2#2 vs siPHLDB2#2 + OE PHLDB2, Figure 4A,B). Similar results were observed in A‐498 cells. (Figure 4D‐F). To further explore the molecular mechanisms of how PHLDB2 decreased VM formation, we examined several VM‐related genes (AKT, MMP9, Vimentin and ERK1/2) by Western blot. It turned out that L‐2HG treatment increased ERK1/2 phosphorylation. In contrast, other relevant pathways associated with VM formation such as MMP9, Vimentin and AKT had no significant change upon L‐2HG treatment (Control vs L‐2HG, Figure 5A). Similarly, reducing the expression of PHLDB2 also increased the ERK1/2 phosphorylation (siNC vs si#1, si#2; Figure 5B). In addition, the effect of PHLDB2 knockdown on ERK1/2 phosphorylation was reversed by restoring PHLDB2 expression (si#2 vs si#2 + OE, Figure 5C). Although statistically significant differences for ERK1/2 phosphorylation were not obtained caused by the limited number of repeated experiments, the change trend of ERK1/2 phosphorylation in each group was consistent (Figure S4A, S4B, S4C and S4D). Consistent with a previous study,^47^ we also treated RCC cells (786‐O and A‐498) with U0126 (1 μM), an effective drug that inhibited ERK phosphorylation, and observed obvious reduction in VM formation compared to the control group (Figure 5D). Overall, these results suggested that L‐2HG functioned through reducing the expression of PHLDB2 and activating the ERK1/2 pathway to alter RCC VM formation (Figure 5E).

## 4 DISCUSSION

In our study, we showed that L‐2HG was elevated in RCC consistent with previous study.^10^ We also demonstrated that L‐2HG, rather than R‐2HG, contributed to the VM formation through reducing the mRNA and protein levels of *PHLDB2*. Our findings provide a new perspective on the important role of L‐2HG in RCC.

2HG is the by‐product of ongoing cellular metabolism produced by enzymes such as fumarate and succinate. In recent years, these oncometabolites were increasingly associated with many tumors including brain tumors,^20^ acute myeloid leukemia,^21^ colorectal cancer,^22^ and head and neck squamous cell carcinoma.^48^ Cheng et al measured the contents of D‐2HG and L‐2HG and observed a 30‐fold increase of L‐2HG in RCC tissues compared to adjacent normal tissues.^15^ Similarly, we also found that L‐2HG was dominant and elevated in RCC compared to the adjacent tissues. More evidence suggested that L2HGDH deficiency was the cause of the elevated level of L‐2HG.^10^, ^49^ Our data also supported the hypothesis that L2HGDH reduced the cellular level of L‐2HG, although there were some other factors such as hypoxia and MDH enzymes implicated in L‐2HG accumulation.^12^ Nevertheless, mechanisms of L‐2HG accumulation in RCC warrant further investigation.

A previous study showed that 2HG activated HIF1α and/or VEGF signaling to stimulate the angiogenesis.^50^ Sunil et al reported L‐2HG as an oncometabolite promoting a migratory phenotype in RCC.^49^ However, the role of L‐2HG in VM formation remained unknown. In our study, we showed that L‐2HG promoted VM in RCC cell lines and that diminishing the L‐2HG via L2HGDH transfection inhibited VM formation. The RCC tissues with high L‐2HG levels also exhibited more VM. Collectively, these findings suggested that the oncometabolite L‐2HG produced by RCC was indeed associated with VM formation.

Our transcriptome analysis revealed that many relevant genes changed after L‐2HG treatment. Through KEGG analysis of these DEGs, we found that many tumor‐relevant pathways were involved, including apoptosis, cell cycle, phosphatidylinositol signaling and AMPK signaling. Furthermore, we found that *PHLDB2* at both mRNA and protein levels were significantly downregulated in several RCC cell lines after L‐2HG treatment. We also observed that PHLDB2 expression was lower in RCC tumor tissues and associated with the OS rate through a Kaplan‐Meier analysis of the TCGA data. These characteristics suggested that PHLDB2 probably played an important role in RCC, although PHLDB2 function was little known. Our data showed that reducing the expression of PHLDB2 led to more VM, which was consistent with L‐2HG treatment. More importantly, restoring the PHLDB2 expression reversed the VM phenotype. Collectively, these results suggested that the L‐2HG probably regulated VM formation through PHLDB2. However, whether PHLDB2 coordinated with other genes to active the ERK1/2 pathway and induce VM formation needs further investigation.

Antiangiogenic therapy is an effective strategy in RCC treatment, especially to slow the progression of tumors. However, the benefits can be limited and most patients develop acquired resistance within a period of treatment. Because of this, as an alternative neovascularization pathway, VM should not be ignored for antiangiogenic therapy. Previous studies demonstrated that TR4, Vimentin and MMP9 induced VM in RCC.^51^, ^52^, ^53^ Our data add the evidence that the oncometabolite L‐2HG can contribute to VM formation. Therefore, targeting L‐2HG is a novel strategy for RCC patients especially with high L‐2HG levels. In addition, some small molecules targeting 2HG have been approved for glioma and leukemia.^54^ AG‐221, a potent and specific inhibitor of mutant *IDH2* that leads to the production of R‐2HG, has already shown significant survival benefits in aggressive leukemia.^55^, ^56^ However, targeting L‐2HG for RCC patients' survival benefits requires more preclinical research.

The present study had limitations. The number of patients involved was insufficient to analyze the relationship between L‐2HG and OS rate. In addition, other potential mechanisms of VM formation caused by the L‐2HG, such as redox metabolism, were not examined. Our future work would involve more patients and confirm the relationship between L‐2HG and prognosis. Further studies of the molecular pathways that regulate 2HG‐induced VM formation are our future research goal.

## CONFLICT OF INTEREST

The authors declare no conflict of interest.

## ETHICS STATEMENT

The present study was approved by Ethics Committee of Sir Run Run Shaw Hospital, School of Medicine, Zhejiang University (number: 20190211‐67). Written informed consent was obtained from the patient for publication of this study.

## Supporting information


**Appendix** **S1**: Supporting Information

## Data Availability

TCGA datasets are available on the UCSC Xena platform (https://xena.ucsc.edu/) and through Wanderer (http://maplab.imppc.org/wanderer/). The RNA‐seq data is deposited to National Genomics Data Center in China for public release (HRA000469). Further details and other data that supports the findings of this study are available from the corresponding authors upon request.
