# Supplementary material for: Oncometabolite L‐2‐hydroxyglurate directly induces vasculogenic mimicry through PHLDB2 in renal cell carcinoma
Source: Int J Cancer. 2021 Jan 15;148(7):1743–55. doi: 10.1002/ijc.33435 (PMC7986127; doi:10.1002/ijc.33435)
Supplement: Supplementary file 1 — Appendix S1: Supporting Information [file IJC-148-1743-s001.pdf]

# Oncometabolite L-2-Hydroxyglurate Directly Induces Vasculogenic Mimicry Through PHLDB2 in Renal Cell Carcinoma

Huan Wang, Liya Wang, Qiming Zheng, Zeyi Lu, Yuanlei Chen, Danyang Shen, Dingwei Xue, Minxiao Jiang, Lifeng Ding, Jie Zhang, Haiyang Wu, Liqun Xia, Jun Qian, Gonghui Li and Jieyang Lu

**Table of contents**

--Supplementary Materials & Methods
--Supplementary Tables 1-2
--Supplementary Figures 1-4

**Supplementary Materials and Methods**

**RNA-seq read mapping**

The Illumina reads (150 bp paired end) were mapped to the annotated genome of GRCh37(hg19) using HISAT2. Approximately 38~55 million 150bp paired-end total read per sample were obtained. To ensure the quality of information analysis, total reads were trimmed to remove any remaining adapter sequences or low-quality reads by TrimGalore (http://www.bioinformatics.babraham.ac.uk/projects/trim_galore/). Then the clean reads were mapped to the annotated genome with parameters. Then FPKM was calculate with the reference genome by Stringtie strategy for further analysis. Sequencing statistics per sample was showed in supplementary Table 2.

Supplementary Table 1: Patient characteristics from TCGA datasets

| Variables | | Case number (N=517) or mean (range) |
|---|---|---|
| Gender | Male | 336 |
| | Female | 181 |
| Age(years) | | 60.64（34-90） |
| Stage | I | 266 |
| | II | 54 |
| | III | 121 |
| | IV | 76 |

Supplementary Table 2: RNA-seq statistics

| Sample | Total reads | Clean reads | Mapped reads | Mapped rate (%) |
|---|---|---|---|---|
| 786-O | 56316634 | 55915234 | 54233628 | 96.99 |
| 786-O+L-2HG | 38540642 | 38277802 | 37048773 | 96.79 |
| A-498 | 53859884 | 53471064 | 51840938 | 96.95 |
| A-498+L-2HG | 55105450 | 54698764 | 52972770 | 96.84 |

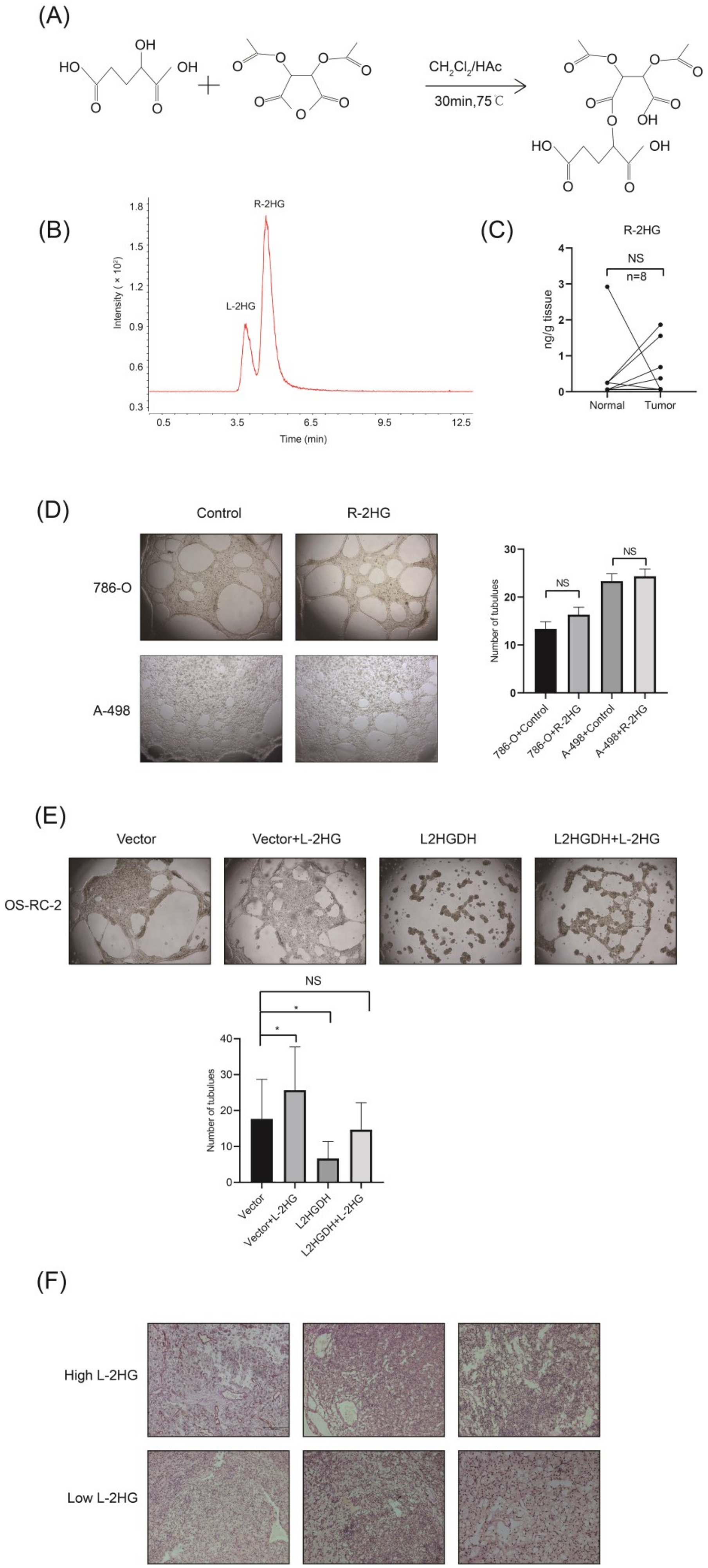
(A)
OH
HO
OH
O
O
CH2Cl2/HAc
30min,75℃
(B)
R-2HG
L-2HG
Intensity ( × 10²)
1.8
1.5
1.2
0.9
0.6
0.3
0.5
3.5
6.5
9.5
12.5
Time (min)
(C)
R-2HG
NS
n=8
ng/g tissue
4
3
2
1
0
Normal
Tumor
(D)
Control
R-2HG
786-O
A-498
Number of tubulues
30
20
10
0
NS
NS
786-O+Control
786-O+R-2HG
A-498+Control
A-498+R-2HG
(E)
Vector
Vector+L-2HG
L2HGDH
L2HGDH+L-2HG
OS-RC-2
NS
*
*
40
30
20
10
0
Number of tubulues
Vector
Vector+L-2HG
L2HGDH
L2HGDH+L-2HG
(F)
High L-2HG
Low L-2HG

**Figure S1**

(A) Reaction scheme for the derivatization of 2HG. DATAN and 2HG was processed in the $CH_2Cl_2$ and HAc in 75℃ for 30min.

(B) Standard solutions of L-2HG and R-2HG were analyzed by LC-MS/MS. Two well resolved peaks for the derivatized L-2HG and derivatized R-2HG were recorded.

(C) R-2HG level was measured by LC-MS/MS in the RCC tissues and adjacent normal tissues, n=8 NS= no significant.

(D) Images（40X） of VM in 786-O and A-498 cells after R-2HG treatment for 24h. VM tubes were quantified in the right panel. NS=no significant.

(E) Images（40X） of VM in OS-RC-2 cells after L-2HG treatment or L2HGDH plasmid transfection. Quantification of VM including three kinds of cell lines (786-O, A-498, OS-RC-2) for each experimental group was presented in the bottom. *$p<0.05$, NS=no significant.

(F) The presence of VM in tumor tissues (High L-2HG vs Low L-2HG). PAS positive and CD34 negative was considered the VM channel. The quantification data was in the Fig1E.

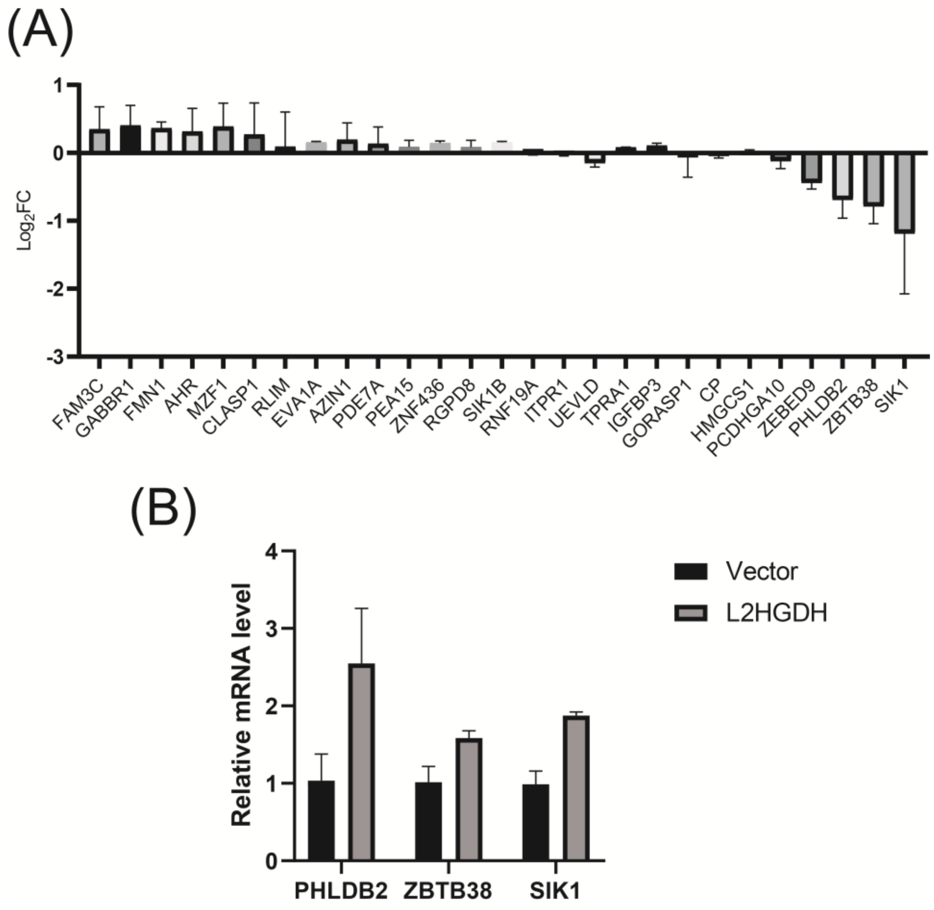


**Fig S2**

(A) In 786-O cells， the mRNA level of DEGs was confirmed via qPCR. The data was shown with the $Log_2FC$ (fold change). SIK1, ZBTB38 and PHLDB2 were the most down-regulated genes.

(B) In 786-O cells, PHLDB2, ZBTB38, SIK1 relative mRNA level increased after L2HGDH plasmid transfection.

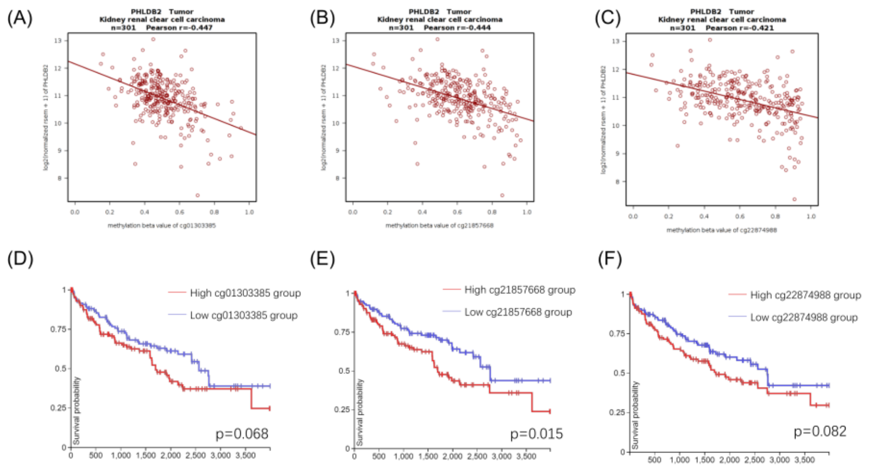


**Figure S3**

**Correlation of methylation with PHLDB2 expression and OS of RCC.**

(A, B, C) Pearson correlation of cg01303385, cg21857668 and cg22874988 with PHLDB2 expression. (D) Kaplan-Meier analysis of overall survival (OS) in RCC patients with cg01303385 level (p=0.068). (E) Kaplan-Meier analysis of OS in RCC patients with cg21857668 level (p=0.015). (F) Kaplan-Meier analysis of OS in RCC patients with cg22874988 level (p=0.082)

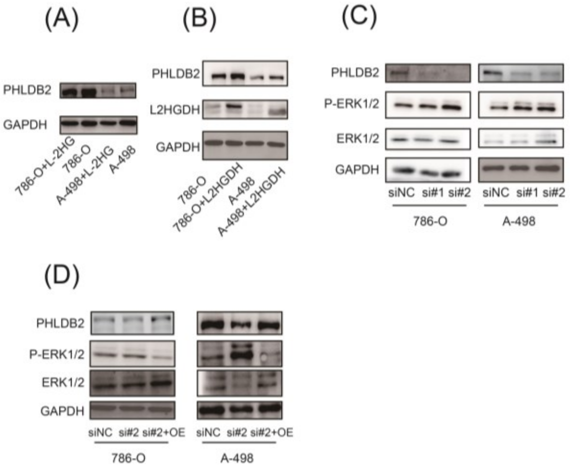


**Fig S4**

(A) Western blot analysis to compare the PHLDB2 expression for 786-O and A-498 cells after L-2HG treatment.

(B)Western blot analysis to compare the PHLDB2 expression for 780-O, 786-O+L2HGDH transfection, A-498, A-498+L2HGDH transfection.

(C)Western blot analysis to compare the ERK1/2 phosphorylation for siNC, siPHDB2#1, siPHLDB2#2 in 786-O and A-498 cells.

(D) Western blot analysis to compare the ERK1/2 phosphorylation for siNC, siPHDB2#2, siPHLDB2#2+ OE PHLDB2 in 786-O and A-498 cells.
